# Effects of StereoBiCROS on Speech Understanding in Noise and Quality of Life for Asymmetric Sensorineural Hearing Loss

**DOI:** 10.3390/audiolres15060176

**Published:** 2025-12-16

**Authors:** Morgan Potier, Arnaud Noreña, Fabien Seldran, Mathieu Marx, Stéphane Gallego

**Affiliations:** 1 CNRS UMR 7260, Laboratoire de Neurosciences Cognitives (LNC), Aix-Marseille, Université Centre Saint-Charles, 3 Place Victor Hugo, 13331 Marseille, France; 2 Laboratoire d’Audiologie Clinique (LAC), Hôpital Privé du Grand Narbonne, 3 Rue du Professeur Christiaan Barnard, 11100 Narbonne, France; 3 Laboratoire Audition Conseil Gratte-Ciel, 40, Rue Michel Servet, 69100 Villeurbanne, France; 4 CNRS UMR 5549, Faculté de Médecine Purpan, Centre National de la Recherche Scientifique, 31000 Toulouse, France; 5 Institut des Sciences et Technologies de Réadaptation (ISTR), Université de Lyon, 8 Avenue Rockefeller, 69008 Lyon, France

**Keywords:** asymmetric sensorineural hearing loss (ASNHL), hearing aid, contralateral routing of signal (CROS), stereophonic bilateral contralateral routing of signal (StereoBiCROS), cochlear implant

## Abstract

**Background and Aim**: Asymmetric sensorineural hearing loss is difficult to rehabilitate acoustically. Bilateral amplification may induce binaural interference, while CROS/BiCROS systems provide benefit only when the speech signal reaches the poorer ear. A hybrid approach combining CROS strategy with bilateral acoustic amplification, called Stereophonic Bilateral Contralateral Routing of Signal—StereoBiCROS—has recently emerged. **Methods**: A one-month home trial was conducted with hearing aids programmed in three listening modes: Stereophonic, BiCROS, and StereoBiCROS. Speech-in-noise perception was assessed in dichotic and reverse-dichotic conditions. Speech recognition thresholds were derived using logistic regression. Daily mode usage was extracted from datalogging. Pre/post subjective benefit was evaluated using the SSQ-15 and SF-12. **Results**: Eighteen participants (mean age 70.7 ± 8.2 years) used the devices 12.4 ± 1.6 h per day, predominantly in StereoBiCROS mode (76.9 ± 24.2%). In the dichotic condition, this mode yielded the best speech-to-noise ratio (0.96 ± 2.74 dB; *p* < 0.0001), outperforming unilateral rerouting (3.00 ± 2.05 dB; *p* = 0.001) and bilateral amplification (5.16 ± 1.31 dB; *p* = 0.001). In the reverse-dichotic condition, only bilateral amplification provided a non-significant improvement (3.08 ± 1.38 dB), whereas the other modes deteriorated intelligibility. SSQ-15 total and subscale scores significantly improved after one month, while SF-12 scores did not change. **Conclusions**: StereoBiCROS stimulation appears to be a promising acoustic alternative for improving speech intelligibility in noise and patient-reported outcomes in asymmetric sensorineural hearing loss. Further research is required to identify the most responsive audiological profiles.

## 1. Introduction

Patients with asymmetric sensorineural hearing loss (ASNHL), defined as unaidable hearing loss in the poorer ear and hearing loss in the better ear, report discomfort in daily living, from both an audiological and psychosocial point of view [1,2]. The deficit produced by ASNHL is primarily related to the disruption of binaural hearing and the loss of the benefits provided by binaural mechanisms, which include the following:

- The elimination of the head shadow effect, which is the most significant phenomenon and corresponds to the amputation of an auditory hemifield [3,4,5];
- The disappearance of the squelch effect, which corresponds to the segregation of speech from background noise [4,6,7,8,9];
- The loss of the summation effect (also called “binaural redundancy”), which results from the addition of the signal presented to both ears [4,5,9];
- The reduced ability to localize sounds in the horizontal plane based on interaural time difference (ITD) and interaural level difference (ILD) [10,11].

Consequently, the near or total loss of hearing in one ear leads to great difficulty recognizing speech when the signal arrives at the poorer ear, reduced intelligibility in noise or reverberant environments [12,13], and difficulty localizing sounds in the horizontal plane [10,14,15]. Finally, ASNHL results in significant listening effort in most everyday situations [16,17,18,19,20,21]. A number of authors also report socio-behavioral consequences, difficulties in daily activities, and thus a decrease in global Quality of Life (QoL) and social interactions [20,22,23,24,25]. In addition to these difficulties, ASNHL patients also suffer from several disabling ancillary symptoms, often little or poorly evaluated [26], such as tinnitus in their poorer ear [27,28], hyperacusis [29], or balance/posture disorders.

To address some of the negative effects of the acoustic head-shadow in patients with ASNHL, hearing rehabilitation can be achieved with a cochlear implant (CI) on the poorer side or a hearing aid (HA). Currently, the gold standard is undeniably the CI [21,30,31,32,33,34,35]. However, this solution may further destroy any residual hearing or vestibular function in the impaired ear, carries surgical risks, and the financial cost is relatively high [36,37,38,39,40]. As standard acoustic amplification by HAs shows real limitations in terms of results [41], an older alternative strategy to restore hearing and also to improve the awareness of sounds for the poorer ear is to reroute signals by air conduction (AC) or bone conduction (BC) to the contralateral side (the better ear). Historically, Fowler (1960) was the first to introduce the contralateral routing of signals (CROS) to help alleviate the problems associated with the amputation of an auditory hemifield. CROS rehabilitation by AC was described in the 1960s [42,43]. This non-surgical management option consists of an HA worn on the poorer ear containing a microphone and a transmitter. This HA transmits the acoustic signal to a receiver in another HA worn on the better ear. When hearing loss is also present in the better ear, a bilateral contralateral routing of signal (BiCROS) approach is utilized. While CROS reroutes signals to the contralateral (non-impaired) ear, a BiCROS system not only allows for the transmission of sounds arriving at the poorer ear to the better ear but also provides amplification to the better ear itself. The CROS device was developed for patients with unilateral sensorineural hearing loss (USNHL), while the BiCROS device was developed for patients with ASNHL. While early devices used a hardwire connection to transmit the acoustic signal from the poorer to the better ear, the technology now allows wireless connectivity between both ears [44].

Although these alternatives are currently considered standard care by the audiology community, there is controversy regarding the benefits of CROS or BiCROS systems. Recent literature [21] indicates the limitations of these systems in specific listening situations. Many studies [45,46,47,48,49,50,51] have shown that CROS or BiCROS devices are successful in quiet conditions and improve the signal-to-noise ratio (SNR) in some noisy environments, particularly in the dichotic condition (when speech is presented to the poorer ear side, and noise is presented to the better ear side (receiver side)). However, a significant decrease in speech intelligibility performance was observed by other authors [52,53,54,55] in the reverse-dichotic condition (when noise is presented to the poorer ear side (transmitter side) and speech is presented to the better ear side (receiver side)). These results are not surprising because the noise (from the poorer ear) is amplified and transferred to the better ear, becoming audible through the aidable ear and interfering with speech presented to the good ear. In his systematic review and meta-analysis from 2016, Kitterick et al. [21] suggest that rerouting devices provide benefits to speech perception in noise when the SNR is more favorable to the poorer ear but degrade speech perception when the SNR is less favorable to the poorer ear. They specify that there is an absence of evidence for any effect of rerouting signals on speech perception when the SNR is similar for both ears [21].

Indeed, this type of equipment has been qualified as “mono-pseudo-stereophonic” [56]: “pseudo” because although the sound capture is bilateral, the stimulation remains unilateral. In other words, while sound capture on the impaired side can offer improved audibility, CROS or BiCROS systems do not restore stereophony, which requires two functional ears to allow central processing of information and thus enable binaural mechanisms [57]. These solutions only partially restore hearing function, and French authorities have not yet recommended any official guideline [40].

**Figure 1:**
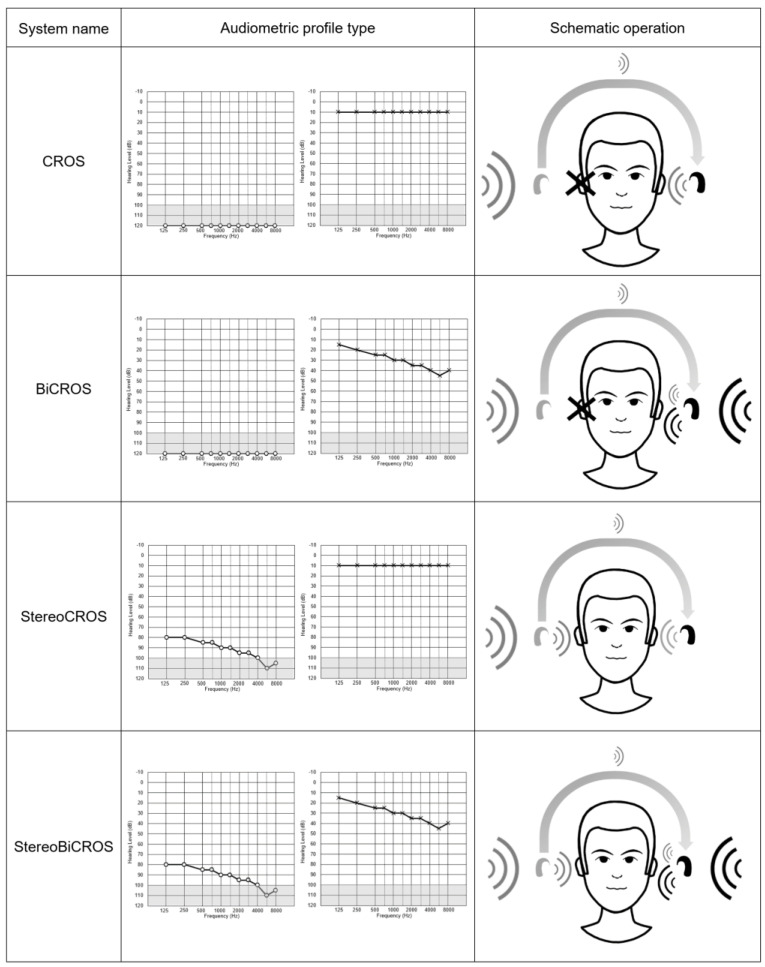
Summary and comparison of different air-conduction CROS systems. For each solution, the typical audiometric profile and a schematic representation of its operation are listed. In these examples, the right ear is represented as the poor ear (schematized by a cross for single-sided deafness or SSD), while the left ear is the better ear.

In view of these limitations, in cases of USNHL or ASNHL, HA manufacturers have recently proposed a new stimulation mode that combines BiCROS with additional amplification of the poorer ear. This option is now available in some Has, and this new mode of stimulation has been called StereoBiCROS [58] for stereophonic bilateral contralateral routing of signal, or TriCROS [58,59] by some audiologists. Indeed, in addition to stimulating the better ear with BiCROS, the StereoBiCROS device is able to appropriately stimulate the USNHL side (residual hearing) with additional amplification. Figure 1 summarizes the different signal rerouting CROS systems according to the audiometric profiles. For the remainder of this paper, we will use the term StereoBiCROS, which is more appropriate than TriCROS to describe this mode of stimulation. The idea of combining the two types of stimulation (CROS and traditional binaural amplification) is not truly new, and to our knowledge the first article referring to this solution dates back to 1990 [60]. This system was named BiCROS-PLUS by the authors and used three microphones: two on the unaidable ear (one for that side, and one to transmit to the contralateral side) and the third for the better ear. BiCROS-PLUS hearing systems were achieved by adding an in-the-canal (ITC) instrument in the poorer ear while retaining the wearer’s existing BiCROS eyeglass hearing instrument. In their original article, the authors report a significant benefit in these patients and describe better speech clarity, significant improvements in localization, and speech recognition in noise. Nevertheless, in this study, BiCROS-PLUS was tested in a very small number of ASNHL patients (N = 6), and the authors do not provide precise quantitative results.

Among the recent investigations into StereoBiCROS stimulation, Potier et al. (2023) examined its impact on tinnitus-related discomfort in ASNHL populations accompanied by bothersome tinnitus [58]. Their findings indicate a significative reduction in tinnitus discomfort comparable to that reported by CI.

The following year, a second study [59] evaluated the benefits of a StereoBiCROS solution for speech understanding in noisy environments. In their work, the StereoBiCROS system (referred to as *TriCROS*) was compared with conventional CROS or BiCROS fittings across several outcomes, including speech-in-noise recognition, spatial localization, and self-reported benefit using the abbreviated profile of hearing aid benefit (APHAB) questionnaire. Although no significant improvement in sound localization was observed, APHAB results indicated enhanced performance in the Ease of Communication, Reverberation, and Background Noise subscales. Moreover, StereoBiCROS provided a statistically significant advantage in speech comprehension in the presence of background noise compared to CROS or BiCROS systems (mean improvement: 1.26 dB; *p* < 0.021). A methodological limitation of the study lies in the use of a single loudspeaker configuration for speech-in-noise testing (French Matrix test), with both speech and noise presented from the same frontal loudspeaker positioned 1 m from the participant. The authors note that future work should explore dichotic test configurations, as commonly implemented in studies evaluating traditional HA fittings. Our study aims to evaluate StereoBiCROS stimulation on ASNHL patients never fitted with HAs and to determine if this new solution improves their QoL and speech intelligibility in noise compared to BiCROS or Stereophonic stimulation. We hypothesize that StereoBiCROS stimulation, by combining the advantages of BiCROS and Stereophonic mode (i.e., the benefits of binaurality), could be the most effective and comfortable stimulation for ASNHL patients. In the present work, to evaluate the performance of speech understanding in noise, we deliberately chose to use both the dichotic and reverse-dichotic conditions—of which the latter is known to be the most difficult for this population—in order to place the patients in a complex situation for which, as studies on BiCROS have shown, their performance is most affected.

## 2. Materials and Methods

### 2.1. Study Design

This work was conceived as a prospective study with repeated measures. Measures were carried out twice: once before the HA fitting (D0), and once one month after the use of HAs (D30). The aim of the study was to investigate the objective and subjective hearing outcomes with this new acoustic rehabilitation system, allowing StereoBiCROS stimulation in a population of ASNHL patients who had never been fitted before. Devices were loaned to the patients, and they were tested at home for a 1 month trial period. We proposed three different programs to each patient: the Stereophonic program, which presented conventional bilateral amplification; the BiCROS program, where only the better ear was amplified, and signals arriving at the poor ear were rerouted to the contralateral ear (i.e., the better ear); and finally the StereoBiCROS program, which was a combination of the two previous techniques, where both ears were amplified, and part of the signals arriving at the poor ear was rerouted to the better ear. Depending on their perception, the patient was free to choose between these three programs the one that gave them the best feeling regarding hearing loss compensation and comfort. To provide a customized rehabilitation strategy, we also assessed the relationship between HA benefit and personal preferences. Datalogging was activated and memorized the daily number of hours (per day) of use for each program and the distribution of use of the three programs, expressed as a percentage.

The present study compares the performances of pseudo binaural benefits of speech perception in noisy conditions between the Stereophonic, BiCROS, and StereoBiCROS programs. This study was carried out in accordance with the Declaration of Helsinki (2013) and was approved on 23 May 2019 by the West 6 Personal Protection Committee (CPP N° 11555-DM2), the French equivalent of an ethics committee. All patients volunteered and provided written informed consent before their participation in the study.

### 2.2. Patients

Nineteen patients suffering from ASNHL (mean threshold 500, 1000, 2000, 4000 Hz dB HL) were included in this study. Their ASNHL had been stable and present for more than 6 months. Patients who had psychiatric or cognitive co-morbidities were excluded from the study. They were adults, French-speaking, and had never worn any HA before starting the study. The patients were recruited at the “Laboratoire d’Audiologie Clinique” (Narbonne, France). They all volunteered and gave their informed consent to participate in this study. Only one patient decided to drop out of the study due to test difficulty and fatigability.

The inclusion criteria included being over 18 years old and being a native French speaker with ASNHL diagnosed more than 6 months ago. In addition, only patients who had never worn HAs (stereophonic or BiCROS) were included in this study. Patients with conductive hearing loss were also excluded from this study.

The final patient sample was composed of 18 patients: 10 women (55%) and 8 men (45%), aged from 54 to 83 years old, with a mean age of 70.7 ± 8.2 years. In total, 10 Left-ASNHL and 8 Right-ASNHL patients joined the study, with the following etiologies: 12 cases of sudden sensorineural hearing loss, 4 cases of deafness due to acoustic trauma or resulting from working in a noisy environment, and 2 cases of deafness consecutive to an ENT history (infections, chronic otitis…).

Descriptive information regarding the population is provided in Table 1.

### 2.3. Hearing Evaluation

Audiological assessments were carried out in a double-walled soundproof room (ISO 8253-2:2009; Acoustics—Audiometric test methods—Part 2: Sound field audiometry with pure-tone and narrow-band test signals. International Organization for Standardization: Geneva, Switzerland, 2009). To test its accuracy, the equipment was calibrated before starting the tests (ISO 389-1:2017; Acoustics—Reference zero for the calibration of audiometric equipment—Part 1: Reference equivalent threshold sound pressure levels (RETSPLs) for pure tones and supra-aural earphones. International Organization for Standardization: Geneva, Switzerland, 2017). After otoscopic examination of the external auditory meatus and tympanic membrane, we performed pure-tone audiometry with TDH39 headphones, in compliance with the ISO 8253 standard (ISO 8253-1:210; Acoustics —Pure tone air conduction threshold audiometry for hearing conservation purposes. International Organization for Standardization: Geneva, Switzerland, 2010). Both tonal audiometry by air conduction (AC) at frequencies 0.25–8 kHz and another by bone conduction (BC) (B71—Radioear Corporation, New Eagle, Ann Arbor, MI, USA) at frequencies 0.5–4 kHz were performed. The hearing thresholds were measured with warbled pure tones, following the Hughson and Westlake manual method [61]: The intensity decreased by 10 dB HL for every accurately perceived tone and increased by 5 dB HL for every missed tone, until the patient twice responded correctly for a single intensity level; this value was then recorded as the threshold for that frequency. If a 10 dB HL gap was found between AC and BC, the patient was excluded from the study. To avoid any transcranial transfer, appropriate acoustic masking was systematically applied in air conduction on the better ear when measuring the performance of the ASNHL ear. Thresholds exceeding the audiometer evaluation limits were set at 120 dB HL. Speech audiometry was then performed in AC using the French Fournier’s dissyllabic word lists [62], with a maximum intensity of 105 dB SPL. The evaluation of the amplification gain—that is, the hearing threshold with the activated hearing aids—was carried out in a free field for each ear. Stimuli were emitted by a loudspeaker positioned in front of the patient’s head, at one meter distance. Even though we could miss existing dead cochlear regions, to avoid standing waves we used warble-tones for each tested frequency, and the faintest sound they could hear with the HA on was determined following the standard clinical procedure. The French Fournier’s dissyllabic word lists for speech audiometry were also presented in a free field, and the patient was asked to repeat the word they heard for each ear, both with and without the HAs. For both free-field hearing thresholds and speech audiometry, during the poorer ear testing, a narrow-band masking sound or a speech masking sound was applied to the better ear using TDH39 headphones to avoid cross-hearing. This masking technique ensured that we obtained the response from the poorer ear.

### 2.4. Audiological Assessment

**Figure 2:**
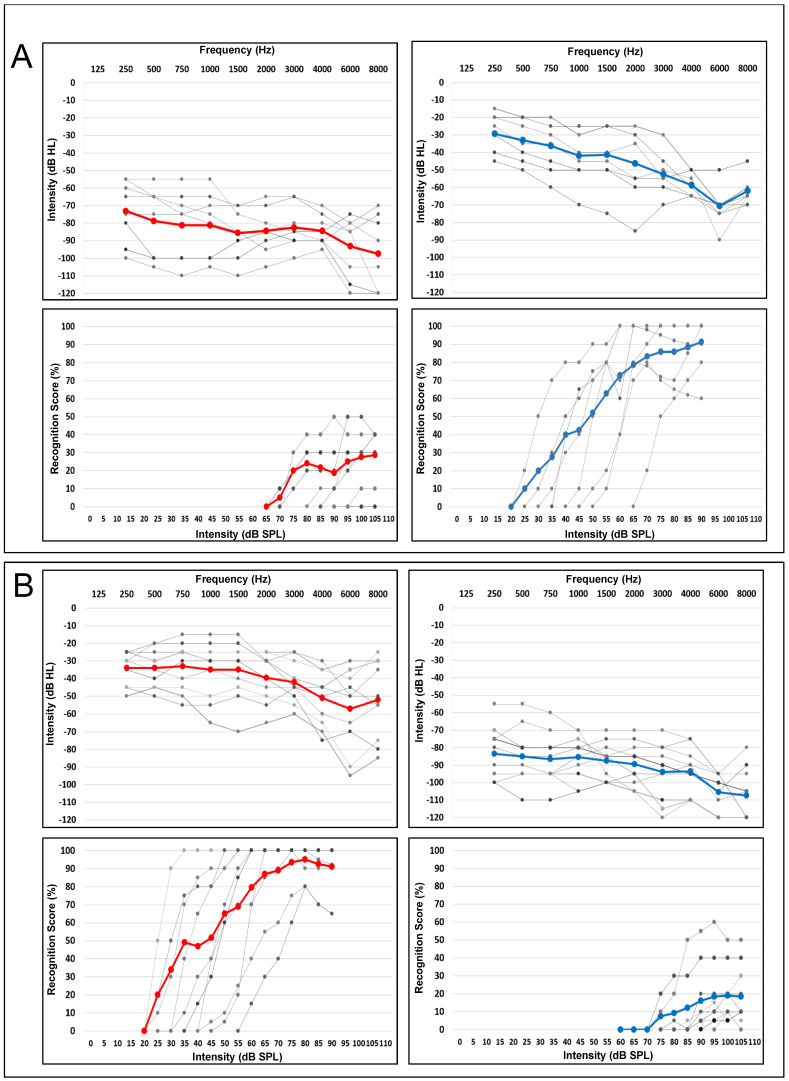
Hearing thresholds and speech audiometry in each ASNHL population. (**A**): Right-ASNHL population (N = 8), PTA_PE_ = 82.2 ± 2.7 dB HL, PTA_BE_ = 45.0 ± 10.7 dB HL. (**B**): Left-ASNHL population (N = 10), PTA_PE_ = 88.4 ± 4.0 dB HL, PTA_BE_ = 40.0 ± 7.8 dB HL. PTA = Pure Tone Average (average of thresholds at frequencies 500, 1000, 2000, 4000 Hz in dB HL).

Figure 2 shows the hearing thresholds and speech audiometry results for the better ear and the poorer ear of each patient (grey lines), as well as the mean pure-tone threshold (red lines for the right ear, blue lines for the left ear). Figure 2A focuses on the Right-ASNHL population, and Figure 2B on the Left-ASNHL population. According to the BIAP classification [63], all patients had severe hearing loss (2nd degree: the pure-tone average at frequencies 500, 1000, 2000, and 4000 Hz was higher than 70 dB HL) on the ASNHL side (Right-ASNHL: 82.2 ± 2.7 dB HL; Left-ASNHL: 88.4 ± 4.0 dB HL). With the better ear, patients exhibited a moderate hearing loss (1st degree: Right-ear = 45.0 ± 10.7 dB HL; Left-ear = 40.0 ± 7.8 dB HL). The mean deafness duration in the population was 193.5 ± 212.4 months. Results obtained by speech audiometry are consistent with the average pure-tone audiometry results: we used the speech recognition threshold (SRT) definition and we did not reach it on the ASNHL side. Mean maximum intelligibility was measured between 20% and 30% depending on which side had ASNHL. The mean maximum intelligibility was 95% for the right better ear and 91% for the left better ear.

For the Right-ASNHL patients, the average tonal prosthetic gain for the poorer ear at 2000 Hz was 21.9 ± 10.3 dB, and the average gain value at 2000 Hz based on hearing loss was 50.5 ± 9.0%. For the Left-ASNHL patients, the average tonal prosthetic gain for the poorer ear at 2000 Hz was 44.0 ± 9.9 dB, and the average gain value at 2000 Hz based on hearing loss was 51.0 ± 8.0%.

### 2.5. HA Fitting

Two HA models were used in this study: Sound SHD-9 and Audeo Belong-90 (Sonova AG, Stäfa, Switzerland). They were the most recent types of HA with 20 channels of settings. HA fitting was carried out under usual test conditions by an experienced hearing instrument specialist (first author). HA fitting was performed using an Aurical Visible Speech system with a wireless SpeechLink 100 binaural measurement unit (Madsen, GN Otometrics, Taastrup, Denmark) to match specified amplification targets using NAL-NL2 methodology and real ear measurement (REM). Ear impressions of each ear were taken to produce tailor-made earmolds to adapt, as much as possible, to the external auditory canal and pinna anatomy. Optimal tightness was sought in the fitted ear (absent venting or 1 mm maximum venting diameter) to deliver maximum acoustic power output, while a more substantial venting (between 1 mm and 2.5 mm) was sought in the better ear, when needed. For optimal results, the ear impression was made with a silicone material with the jaw open and the ear protector placed beyond the second bend. We used a soft earmold/shell material with a long bore for maximum tightness on the ASNHL side (absent venting or 1 mm maximum venting diameter) to deliver maximum acoustic power output, while we focused on providing a more substantial venting (between 1 mm and 2.5 mm) on the side of the better ear. By having the sound bore beyond the second bend, the amplified signal will be placed near the tympanic membrane of the ear canal and be more efficient in sending the amplified signal. The BiCROS system allows a wireless transmission of the complete audio bandwidth (130 Hz–6.0 kHz) from the transmitter device located on the ASNHL side to the receiver device located on the better ear side. To achieve this, manufacturers used inductive transmission technology with digital coding of carrier frequency 10.6 MHz (Harmonised European Standard ETSI 300 330, operation frequency 10.6 MHz, bandwidth 498 MHz, Differential Quadrature Phase Shift Keying (DQPSK) modulation, magnetic field strength −25.5 dB µA/m). During this wireless transmission, the microphone of the transmitting device was set in omnidirectional mode. The volume control was made inactive throughout the duration of the tests to avoid any bias related to an intensity discrepancy. Each patient’s daily HA wearing time was recorded and saved in an online system.

Three different programs were available to the patients: Stereophonic, BiCROS, and StereoBiCROS. The Stereophonic program consisted of conventional bilateral amplification. The BiCROS and StereoBiCROS programs both consisted of rerouting signals coming from the poorer ear to the better ear. In addition, the BiCROS program included amplification only to the better ear side, while the StereoBiCROS program also included amplification of the poorer ear (i.e., bilateral amplification). When the HA was turned on, the StereoBiCROS program was activated as the default program. The programs were ordered as follows, in a loop: Stereophonic, BiCROS, and StereoBiCROS.

Importantly, this configuration was not chosen to prioritize StereoBiCROS, but rather for purely practical reasons linked to device ergonomics and patient usability. Assigning a fixed first program ensured that participants could easily cycle through the three modes using the physical program-change button on the HA—a standard and widely adopted procedure in HA rehabilitation.

Moreover, the order of programs did not constrain or bias patient use. Participants received explicit instructions to systematically switch and test each of the three programs across a broad range of real-life listening environments during the one-month trial period. They were asked to evaluate them based on subjective perception—including comfort, listening effort, and speech intelligibility—without preference suggested for any mode.

To ensure adherence to this instruction, we monitored datalogging information over the entire 30-day trial, allowing objective verification that all programs had been explored. This datalogging analysis was conducted by an experienced clinical audiologist (first author), who confirmed that each participant had effectively tested the three stimulation configurations.

This procedure aligns with established clinical practice, where patients typically change stimulation modes through the device’s button interface, and ensures that all programs were evaluated under ecologically valid.

### 2.6. Speech in Noise Perception

All free-field measurements were carried out in an acoustic cabin with an equivalent level over 1 h of 26 dB(A) and a reverberation time of 0.45 s at 500 Hz. In accordance with the study design, speech perception in noise was tested in an unaided and an aided condition using the French Fournier’s dissyllabic word lists [62], in a male voice (10 dissyllabic words per list) recorded at a 44.1 kHz sampling frequency with a 16-bit quantization range. To increase test sensitivity, we opted for syllabic word counting (20 syllables per list), which allowed us to have a precision level of 5%. For the noise characteristics, we chose a standardized noise, the cocktail-party noise because it is more ecological than white noise and is more like the type of noise encountered daily by patients. This noise consists of the recording of several speakers of different sexes and ages. It is broadband noise, very close to the long-term speech spectrum.

The signals (speech and noise) were presented using two speakers (Siare Alpha-22) positioned laterally from the patient (−90° and +90°), 1 m away and at 1.20 m from the ground (at the patient’s ear height). The first speaker emitted a word list at a fixed intensity of 55 dB SPL, while the other speaker emitted cocktail-party noise, incremented by 5 dB SPL with each new list.

**Figure 3:**
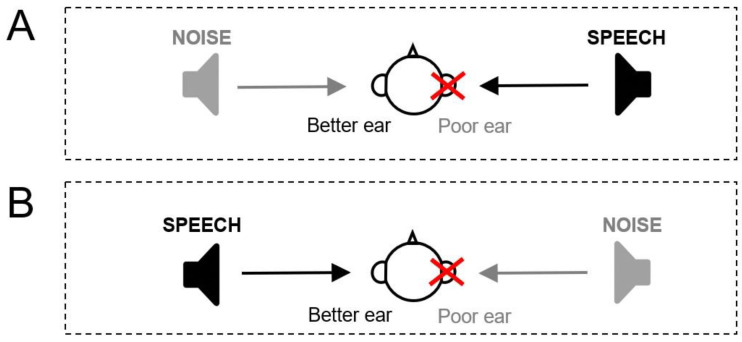
Loudspeaker positioning for speech perception in noise tests. (**A**): Dichotic condition: Speech is emitted to the poor ear (ASNHL side) and noise (cocktail-party) to the better ear. (**B**): Reverse-dichotic condition: Speech is emitted to the better ear and noise (cocktail-party) to the poor ear (ASNHL side). The patient is seated centrally between two loudspeakers positioned laterally, 1 m away from the subject and 1.20 m above the floor (at ear height). The speech signal (French Fournier’s disyllabic word lists) is fixed at an intensity level of 55 dB SPL. The noise (cocktail-party noise) intensity is increased in 5 dB steps for each new list to obtain signal-to-noise ratios (SNRs) ranging from +5 to −15 dB.

We tested patients under two different conditions (loudspeakers positioning is shown Figure 3):

- Dichotic Condition (Figure 3A): Speech is emitted on the ASNHL side and noise to the better ear. Five lists of words are presented at an intensity of 55 dB SPL by varying the noise level from 45 to 65 dB SPL in order to obtain an SNR varying from +10 to −10.
- Reverse-dichotic condition (Figure 3B): Speech is emitted on the side of the better ear and noise on the ASNHL side. Five lists of words are presented at an intensity of 55 dB SPL by varying the noise level from 50 to 70 dB SPL in order to obtain an SNR varying from +5 to −15.

Before the tests were carried out, two training lists were used. The order of the conditions’ presentation (dichotic or reverse-dichotic) to the different SNRs, as well as the program tested (StereoBiCROS, BiCROS, and Stereophonic), were randomized to minimize bias.

### 2.7. Subjective Assessment of Hearing Perception

Before the start of the trials (**D0**) and after 1 month of regular use of HAs (**D30**), each patient completed two questionnaires:

- The Speech Spatial and Qualities of Hearing Scale (SSQ-15) [64], which is the short form French version of the SSQ [19]. This questionnaire is a widely used self-report measure of hearing and has already been translated into several languages [65,66,67,68].

The SSQ measures the impact of hearing loss on different situations in a patient’s daily life. It acts as an intermediate link between the audiological measurement of hearing loss and their social handicap [69]. It is used in many studies assessing the effects of bilateral hearing aids [70,71,72,73], cochlear implants [74,75], or bone-anchored hearing aids [76,77].

The short form version contains 3 subscales (Speech hearing, Spatial hearing, and Hearing quality), each consisting of 5 questions for which the patient is asked to answer on a graduation scale from 0 to 10: 0 means “complete incapacity” and 10 “perfect skill.” The summation of the scores obtained in the 3 parts yields a total score measuring the overall impact of the patient’s hearing loss on their daily life. One of the advantages of the SSQ lies in its ability to measure the impact of rehabilitation by hearing aids in the context of unilateral deafness [70].

- The SF-12 questionnaire [78], which is an abridged and validated version in French of the SF-36 “Short Form Health Survey” [79]. The SF-12 Health Survey questionnaire was originally developed in the United States to provide a shorter alternative to the SF-36, for use in large-scale health measurement and monitoring efforts in which a 36-item questionnaire was too lengthy and in which the focus was on overall physical and mental health outcomes [80]. SF-12 is a questionnaire that measures generic QoL by exploring a patient’s physical, emotional, and social health. It includes 8 dimensions, like the SF-36 (physical activity, life and relationships with others, physical pain, perceived health, vitality, limitations due to mental state, limitations due to physical condition, and mental health). Although non-specific to hearing, it is widely used in medical studies because it allows one to measure the overall health of an individual without considering any specific pathology. The item selection and validation study were carried out in France and in 9 European countries with 9000 people [78]. In its abridged version, it consists of 12 questions to which the patient answers. A score is given for each answer, and adding all the scores together gives two scores (each scored out of 100): the physical health score and the mental health score. In their 1998 publication, Gandek et al. provide a mean score (and standard deviation) by age groups for the physical and mental health scores for each of the countries studied [78]. A score greater than 50 corresponds to an average QoL, a score between 40 and 49 indicates a slight disability, a score between 30 to 39 a moderate disability, and a score less than 30 a severe disability.

To avoid biases that could be linked to the duration of the tests and/or patient fatigue, we did not favor the long versions of these questionnaires.

### 2.8. Statistical Analysis

Throughout this paper, statistical analyses were performed using XLSTAT software (version 2024.3.0 (build 1423), Addinsoft, New York, NY, USA, USA) and are presented as mean standard deviation.

For every testing configuration (dichotic and reverse-dichotic), we used a logistic regression analysis (logit model with likelihood maximization), which allows determining with precision the SRT-50% for every situation. In our study, the SRT-50% is determined by the SNR for which the patient’s performance is decreased to intelligibility.

Then SRTs are compared to each other using a Wald Chi-Squared Test, which allows highlighting the effects of the intensity, the tested conditions, and the programs used (Stereophonic, BiCROS, and StereoBiCROS), and also Cohen’s *d* to estimate effect size. Due to the multiple comparisons between the Unaided, Stereophonic, BiCROS, and StereoBiCROS conditions, the significance thresholds considered in the present study are 0.008, 0.0016, and 0.00016, corresponding to 0.05, 0.01, and 0.0001 divided by 6. The normality of the distribution was assessed by using the Kolmogorov–Smirnov test. For SSQ-scores analysis, we used paired *t*-test comparisons, and we performed the Pairwise Multiple Comparisons test (also called Nemenyi’s test) to assess differences between the utilization time of each program. Differences were considered statistically significant when *p* < 0.05.

## 3. Results

### 3.1. Speech Perception in Noise

**Figure 4:**
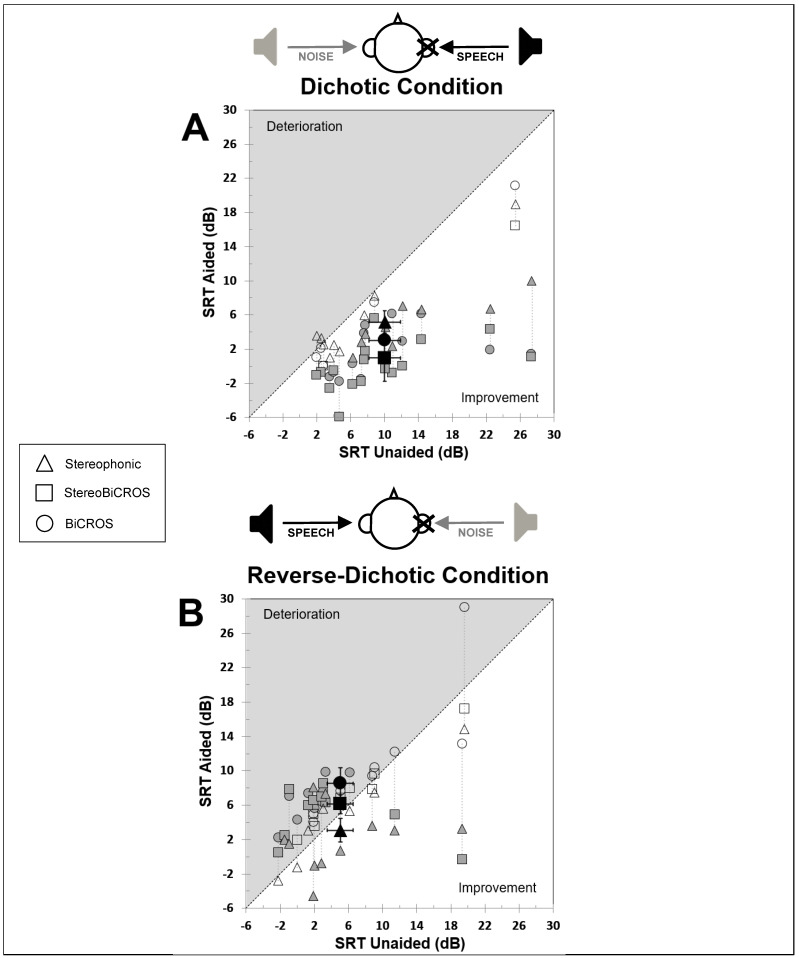
SRT-50% in aided versus unaided conditions. Speech recognition threshold (SRT-50%) in the aided condition for each program plotted against the unaided condition for the dichotic (**A**) and reverse-dichotic (**B**) conditions. In the diagram, the X drawn on the ear indicates the poorer side. The triangles, circles, and squares represent the SRT-50% measured for the Stereophonic, BiCROS, and StereoBiCROS programs, respectively. Small symbols represent individual patient data (empty symbols = non-significant change, grey symbols = significant change), and large black symbols represent the mean value and standard error. Vertical dotted lines connect the results for the same patient. A result located above the diagonal (in the upper half-square, or grey area) indicates a deterioration in performance, while a result below the diagonal (in the lower half-square, or non-grey area) indicates an improvement in performance.

Figure 4 represents, among the 18 patients, the SRT-50% (expressed in dB) obtained for the Stereophonic condition (triangle symbols), the BiCROS condition (circle symbols), and the StereoBiCROS condition (square symbols) depending on the SRT obtained in the unaided condition. Figure 4A represents results for the dichotic condition, and Figure 4B for the reverse-dichotic condition. A result above the diagonal (in the upper half-square = gray area) shows a deterioration of the result, while this result is improved if it is below the diagonal (in the lower half-square = non-gray area).

### 3.2. Dichotic Condition

The SRT-50% mean difference was significantly improved for all programs compared to the unaided condition: 0.96 ± 2.74 dB (*p* < 0.0001; ddl = 17; *d* = 1.16) for StereoBiCROS program, 3.00 ± 2.05 dB (*p* = 0.001; ddl = 17; *d* = 0.94) for BiCROS program, and 5.16 ± 1.31 dB (*p* = 0.001; ddl = 17; *d* = 0.73) for Stereophonic program.

In this condition, 16 patients (88.8%) obtained a significant improvement in their speech perception in noise with the StereoBiCROS program, 13 patients (72.2%) with the BiCROS program, and 9 patients (50%) with the Stereophonic program. With this last program, two patients (11.1%) obtained deteriorating results (non-significant).

**Figure 5:**
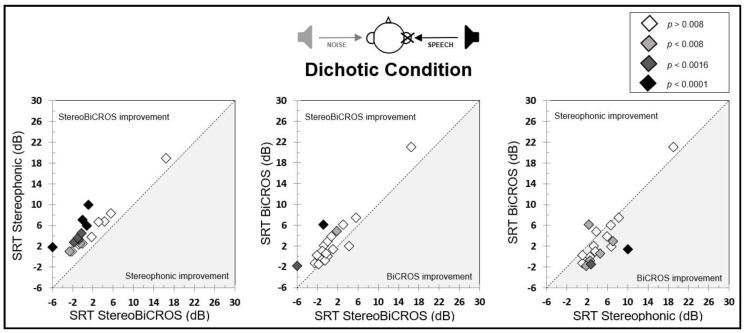
Individuals SRT-50% results for stereophonic, BiCROS, and StereoBiCROS programs compared one-to-one for the dichotic condition. The various symbols represent the various degrees of significance with Bonferroni correction. A result above or below the diagonal shows an improvement for one of the programs.

Individual SRT-50% results for Stereophonic, BiCROS, and StereoBiCROS programs compared one-to-one are shown in Figure 5. Between StereoBiCROS and the Stereophonic programs, 10 patients (55.5%) were significantly improved with the StereoBiCROS program (two *p* < 0.008; four *p* < 0.0016; four *p* < 0.0001). Between StereoBiCROS and the BiCROS programs, three patients (16.66%) were significantly improved with the StereoBiCROS program (one *p* < 0.008; one *p* < 0.0016; one *p* < 0.0001) while only one patient (5.55%) obtained improvement with the BiCROS program (non-significant). Between Stereophonic and the BiCROS programs, six patients (33.33%) were significantly improved with the BiCROS program (four *p* < 0.008; one *p* < 0.0016; one *p* < 0.0001) and three patients were improved with the Stereophonic program, including only one patient (5.55%) significantly (one *p* < 0.008).

### 3.3. Reverse-Dichotic Condition

Compared to the unaided condition, the SRT-50% mean difference was non-significantly improved only for the Stereophonic program (3.08 ± 1.38 dB; *p* = 0.136; ddl = 17; *d* = 0.35) and deteriorated for the other two programs: not significantly for the StereoBiCROS program (6.13 ± 1.1 dB; *p* = 0.460; ddl = 17; *d* = 0.2) and significantly for the BiCROS program (8.53 ± 1.84 dB; *p* < 0.001; ddl = 17; *d* = 0.55).

In this condition, amongst the 18 patients, 4 patients (22.2%) obtained improvement in their speech perception in noise with the StereoBiCROS program (2 significantly), and 14 patients (77.77%) obtained deteriorating results (8 significantly). With the BiCROS program, 1 patient (5.55%) obtained improved results (not significantly), and 16 patients (88.88%) obtained deteriorating results (10 significantly). Finally, with the Stereophonic program, 12 patients (66.66%) obtained improved results (7 significantly), and 6 patients (33.33%) obtained deteriorating results (4 significantly).

**Figure 6:**
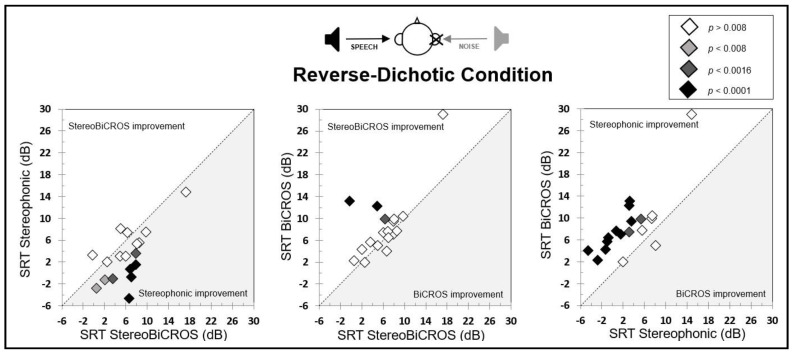
Individuals’ SRT-50% results for stereophonic, BiCROS, and StereoBiCROS programs compared one-to-one for the reverse-dichotic condition. The various symbols represent the various degrees of significance with Bonferroni correction. A result above or below the diagonal shows an improvement for one of the programs.

Individual SRT-50% results for stereophonic, BiCROS, and StereoBiCROS programs compared one-to-one are shown in Figure 6. Between StereoBiCROS and the Stereophonic programs, eight patients (44.4%) were significantly improved with the Stereophonic program (two *p* < 0.008; two *p* < 0.0016; four *p* < 0.0001) and, among ten remaining patients, three patients (16.66%) were improved with the StereoBiCROS program. Between StereoBiCROS and the BiCROS programs, three patients (16.66%) were significantly improved with the StereoBiCROS program (one *p* < 0.0016; one *p* < 0.0001) and, among fifteen remaining patients, five (25.77%) patients were improved with the BiCROS program (only one patient obtained the same scores between StereoBiCROS and BiCROS programs). Finally, between Stereophonic and the BiCROS programs, twelve patients (66.66%) were significantly improved with the Stereophonic program (two *p* < 0.0016; ten *p* < 0.0001) and, among six remaining patients, one patient (25.77%) was improved with the BiCROS program (and only one patient obtained the same scores between Stereophonic and BiCROS programs).

### 3.4. Cost-Effectiveness Ratio of the Conditions

**Figure 7:**
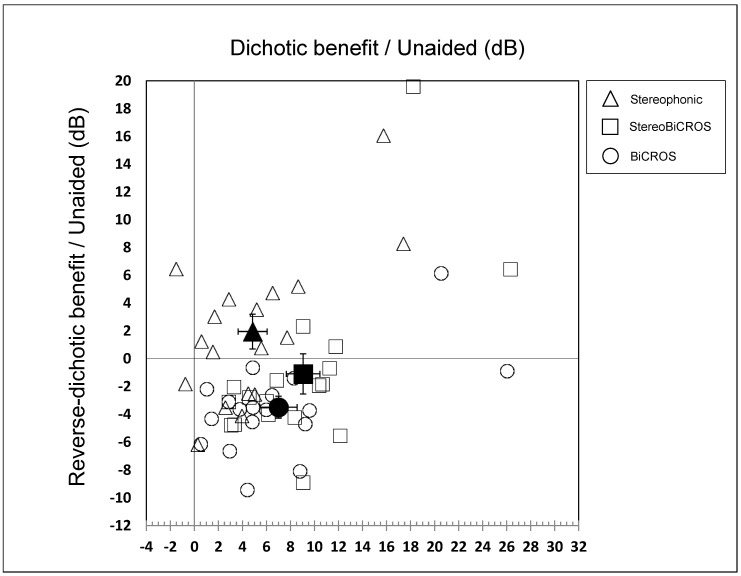
Cost-effectiveness ratio for the different conditions tested (dichotic and reverse dichotic) for each program. The triangle symbols represent SRT-50% measured for the stereophonic program, the circle symbols represent the SRT-50% measured for the BiCROS program, while the square symbols represent the SRT-50% measured for the StereoBiCROS programs. Empty symbols represents individuals data, and filled symbols represent mean values and standard errors.

Figure 7 shows the SRT-50% in the reverse-dichotic condition as a function of the SRT-50% in the dichotic condition for the unaided, Stereophonic (filled triangles), BiCROS (filled circles), and StereoBiCROS (filled squares) conditions. Big symbols represent the mean values, error bars represent the standard errors, and small symbols represent individual data.

Compared to the unaided condition:

- The BiCROS condition provides a mean improvement of 7.0 ± 1.5 dB in dichotic configuration, which cost a degradation of 3.5 ± 0.8 dB in reverse-dichotic configuration.
- The Stereophonic condition provides a mean improvement of both configurations, with 4.8 ± 1.2 dB in dichotic configuration and 1.9 ± 1.2 dB in reverse-dichotic configuration.
- The StereoBiCROS condition provides a mean improvement of 9.0 ± 1.4 dB in dichotic configuration, which cost a degradation of 1.1 ± 1.4 dB in reverse-dichotic configuration.

### 3.5. Questionnaire Evaluation

#### 3.5.1. Speech Spatial and Qualities of Hearing Scale (SSQ-15)

**Figure 8:**
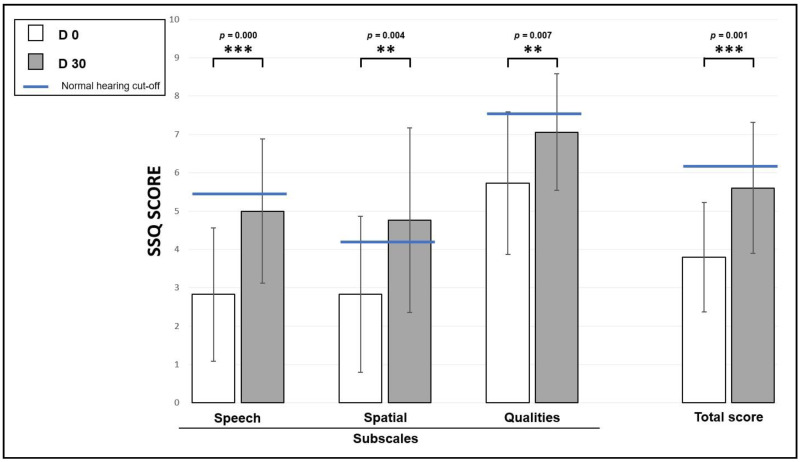
Speech, Spatial, and Qualities of Hearing Scale (SSQ-15) total and subscales scores. Scores were obtained on a 10-point Likert Scale (0 = not at all, 10 = perfectly) to D0 (white bars) and to D30 (grey bars) and represents the mean ± standard error for N = 18 patients. Blue solid lines in each panel express the limit cut-off values for a normal-hearing population of the same age [64]. **/*** Indicates a significant difference.

Figure 8 shows the total mean score (far right of graph) and subscale means organized by SSQ-15 domain (Speech, Spatial, and Qualities) for 18 ASNHL patients. Scores were obtained on a 10-point Likert Scale (0 = not at all, 10 = perfectly) at D0 (white bars) and at D30 (grey bars) and represent the mean ± standard error. A higher value indicates greater perceived ability. The total score demonstrates an improvement in perceived abilities between D0 and the 1-month trial period. Paired *t*-tests showed a significant difference across the sessions in each of the three sub-sections and the total SSQ scores. At D30, total and subscale scores significantly improved compared to D0 scores: Total-score improved from 3.8 ± 1.4 at D0 to 5.6 ± 1.7 at D30 (t = −4.18, *p* = 0.001, ddl = 17), Speech-score improved from 2.8 ± 1.7 at D0 to 5.0 ± 1.9 at D30 (t = −4.66, *p* = 0.000, ddl = 17), Spatial-score improved from 2.8 ± 2.0 at D0 to 4.8 ± 2.4 at D30 (t = −3.34, *p* = 0.004, ddl = 17) and Qualities-score score improved from 5.7 ± 1.9 at D0 to 7.1 ± 1.5 at D30 (t = −3.08, *p* = 0.007, ddl = 17). The limit cut-off values for each domain are represented by the blue horizontal solid lines [64] and represent the norm for a normal-hearing population of the same age as our population (6.14 for Total-Score; 5.45 for Speech-Score; 4.21 for Spatial-Score; and 7.63 for Qualities-Score).

#### 3.5.2. Short Form Health Survey (SF-12)

**Figure 9:**
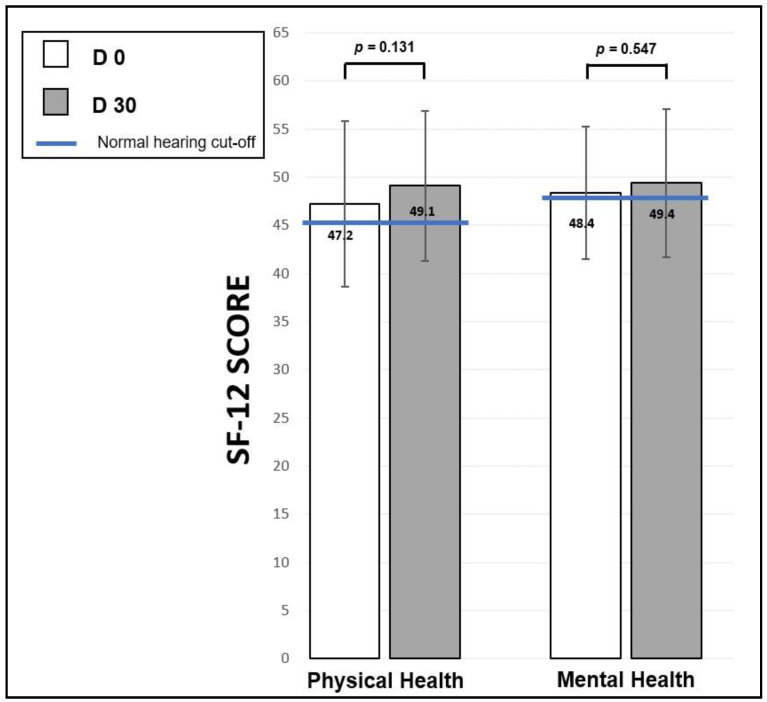
Short Form Health Survey (SF-12) scores. Mean scores ± standard error obtained from the Short Form Health Survey questionnaire at D0 (white bars) and at D30 (gray bars) evaluating the physical and mental health of the 18 patients. Blue solid lines express the limit cut-off values for a French population of the same age as our cohort [78].

Figure 9 shows the mean scores obtained on the SF-12 questionnaire for physical health (left) and mental health (right) of the 18 ASNHL patients. Scores were obtained on a scale from 0 to 100 at D0 (white bars) and at D30 (grey bars) and represent the mean ± standard error. Between D0 and D30, the scores of the two dimensions studied improved, but the *t*-test did not show any significant difference between the sessions in each of the dimensions studied. Indeed, for physical health, the score improved from 47.2 ± 8.6 at D0 to 49.1± 7.8 at D30 (t = −1.58, *p* = 0.131, ddl = 17), while for mental health, the score is improved by 48.4 ± 6.9 at D0 and 49.4 ± 7.7 at D30 (t = −0.61, *p* = 0.547, ddl = 17). The limit cut-off values for each domain for France are represented by the blue horizontal solid lines [78] for the same age as our population (45.7 ± 9.0 for Physical Health and 48.3 ± 9.2 for Mental Health).

### 3.6. HA Utilization

Using datalogging, the average daily usage between each program was significantly different. During the 1-month HA trial, we verified that each of the programs could be tested by patients, and the mean daily usage ranged from 9.5 to 15.0 h per day (12.4 ± 1.6 h/day). This suggests that all the patients used their HAs intensively. If each program was tried out by the patients, the StereoBiCROS program was the most used program (76.9% ± 24.2%). The Stereophonic program was the 2nd-most-used program (18.6% ± 20.5%). Finally, the BiCROS program was the least-used program (4.5% ± 6.7%).

The difference in daily usage between the StereoBiCROS and the BiCROS programs was significant (Nemenyi’s test scored *p* < 10^−4^), as well as the difference between the StereoBiCROS and the Stereophonic programs (Nemenyi’s test scored *p* = 0.043), or as the difference between the BiCROS and the Stereophonic programs (the Nemenyi’s test scored *p* = 0.022). We can estimate the total stimulation time of the ANSHL side was 95.5% ± 6.6% of the time (this corresponds to addition of the stimulation duration of StereoBiCROS and Stereophonic programs).

## 4. Discussion

### 4.1. Summary of the Results

The new StereoBiCROS stimulation was tested on 18 ASNHL patients never fitted with HAs to investigate which amplification program (Stereophonic, BiCROS, and StereoBiCROS) allowed the best objective result (speech recognition performance in noise in dichotic and reverse-dichotic conditions) and also subjective sensation (datalogging and SSQ-15 and SF-12 scores) in their daily life. In the dichotic condition, the SRT-50% mean difference was significantly improved for all programs compared to the unaided condition, but the StereoBiCROS program obtained the best results, followed by the BiCROS program and finally the Stereophonic program. In the reverse-dichotic condition, these results are different: the Stereophonic stimulation is the only program to obtain a non-significant improvement in the SRT-50% mean difference compared to the unaided condition, while the other two programs showed a deterioration in speech intelligibility. Our study also shows that the StereoBiCROS program was subjectively preferred by all patients. Indeed, during the month-long trial, patients used their HAs daily intensively (12.4 ± 1.6 h per day), and they mainly chose the StereoBiCROS program, followed by the Stereophonic and finally the BiCROS program. The results obtained from the questionnaires with HAs (not directly pertaining to any particular program) show a significant improvement in scores in each of the three subscales (Speech, Spatial, and Qualities), as well as the total score of the SSQ-15, while this improvement is not significant for the SF-12 questionnaire.

### 4.2. Effectiveness of Current Prosthetic Solutions

While the results in the literature for rehabilitation by amplification of symmetrical bilateral hearing loss can be considered unanimously good [25,81,82,83], the results are different for the prosthetic rehabilitation of USNHL or ASNHL. In these populations, while hearing correction by bilateral amplification seems logically the most appropriate (reduction of interaural deviations, partial restoration of stereophony, etc.), it does not always provide the expected benefit. Some authors have even observed significantly lower word recognition performance for bilaterally fitted patients compared to the unilateral fitting condition [41]. This could be due to the appearance of “binaural interference,” a phenomenon known since the 1990s and still debated, where the response of the poorer ear seems to harm the contralateral ear [84,85,86,87,88] because of the importance of interaural asymmetry in terms of discrimination. The risk is the abandonment of the HA on the poor ear side, as it brings the least useful information to the central auditory system [89].

In fact, despite older data in the literature demonstrating the advantages of bilateral restoration [90,91], Cox et al. showed that 46% of patients preferred a monaural rather than bilateral fitting [92]. Bishop et al., in their study carried out in 2017 on a series of 22 patients [41], report that almost half of them (41%) decided to give up HAs for medical reasons—specifically, lower performance with two HAs versus just one.

These elements could partly explain why the evolution of the literature as well as clinical practice has gradually abandoned the bilateral prosthetic solution choice in the specific rehabilitation of ASNHL (stimulation of the poor ear) in favor of CROS solutions [21,93]. Indeed, CROS or BiCROS systems are today the most conventionally indicated for the prosthetic rehabilitation of ASNHL. Recently, two literature reviews [21,94] studied the performance of CROS (AC or BC) compared to the unaided condition and synthetically show the following:

- A beneficial effect when the SNR on the side of the poor ear is favorable, i.e., in a dichotic condition.
- A deleterious effect when the SNR on the side of the better ear is favorable, i.e., in a reverse-dichotic condition.

This difference in terms of performance, which is condition-dependent, could explain why a large number of patients who nevertheless meet the criteria for CROS indications (BC or AC) end up rejecting the device by refusing, for example, the implantation of bone-CROS after prosthetic trials in 32% to 69.6% of cases, depending on the study [95]. This contradiction between good prosthetic results in the literature and refusal to wear the device can therefore be explained by the fact that the studies that have evaluated CROS systems have opted, for the most part, for a dichotic condition for evaluating speech-in-noise performance, which is the most troublesome for the patient but the most rewarding from a rehabilitative point of view. Indeed, by using this test condition in noise, the SNR in the patient’s better ear is greatly improved, and thus the CROS results are maximized. On the other hand, in the reverse-dichotic condition, these same devices (by transferring noise to the better ear) degrade the SNR in the better ear, which will have the effect of degrading performance compared to the unaided condition. Furthermore, if CROS devices make it possible to short-circuit the poorer ear by sending useful information back to the better ear, they do not allow for real stereophony. This type of equipment cannot be described as stereophonic because although the capture is done bilaterally, the stimulation remains unilateral.

**Figure 10:**
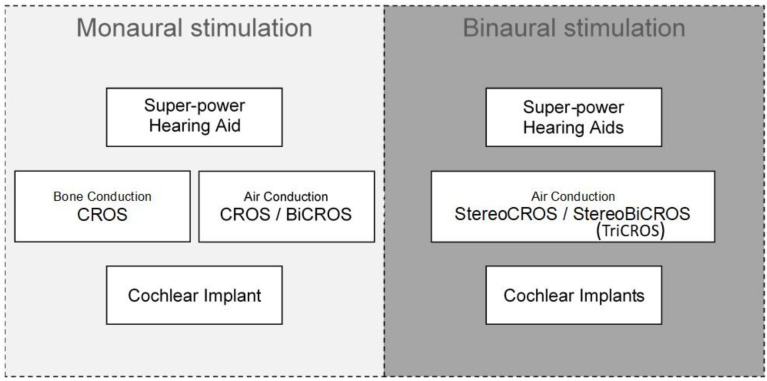
Summary diagram showing the different possibilities for rehabilitation of AHL according to monaural or binaural stimulation. *CROS = contralateral routing of signal, BiCROS = bilateral contralateral routing of signal*.

This is why, in the context of the rehabilitation of USNHL/ASNHL patients, the practitioner is daily led to find compromises, by proposing for example to keep only one HA, or a CROS system (AC or BC), each with their own limitations. The StereoBiCROS hybrid system, combining both bilateral stimulation and also CROS transfer to the better ear, therefore overcomes this compromise: the practitioner does not have to make a forced choice between the two existing modalities. Table 2 summarizes the advantages and disadvantages of the different solutions for the rehabilitation of severe to profound AHL, while Figure 10 is a summarizing diagram of the different rehabilitation possibilities according to monaural or binaural stimulation.

### 4.3. Effects of StereoBiCROS on Speech Perception in Noise

In our study, in the dichotic condition, the SRT-50% with the StereoBiCROS program obtained the best results (0.96 ± 2.74 dB; *p* < 0.0001), then the BiCROS program (3.00 ± 2.05 dB (*p* = 0.001), and finally the Stereophonic program (5.16 ± 1.31 dB (*p* = 0.001).

Although difficult to compare because StereoBiCROS is a hybrid system, our results are fully aligned and consistent with the literature on CROS or BiCROS systems [46,47,48,49,50,51]: in a dichotic condition, the CROS transfer for StereoBiCROS or BiCROS programs allows patients to perform better by improving the SNR in the better ear, unlike the stereophonic program which is de facto limited by the interaural asymmetry and also by the weak residual hearing capacities of the poorer ear.

A new finding is that the results obtained with StereoBiCROS stimulation are less deteriorated in the reversed-dichotic condition (SRT-50% = 6.13 ± 1.1 dB; *p* = 0.460) and therefore less penalizing for the patient than the historical BiCROS stimulation mode (SRT-50% = 8.53 ± 1.84 dB; *p* = 0.000). Naturally, Stereophonic stimulation, by definition unaltered for the reverse-dichotic condition, provides the best (non-significant) result for the SRT-50% mean difference compared to the unaided condition (3.08 ± 1.38 dB; *p* = 0.136).

Indeed, stereophonic stimulation, more natural than StereoBiCROS or BiCROS, has the advantage of not being dependent on the lateralization of the noise in the reverse-dichotic condition. This is why, if this mode has the least benefit in dichotic conditions, it is the only one of the three programs to obtain positive SRT-50% values in the two conditions of the evaluated tests (no degradation of speech intelligibility in noise compared to unaided ears). Kuk et al., in their study of a small cohort of nine adults equipped with BiCROS, show that the negative effect of performance degradation for ASNHL patients due to the CROS-transfer in a dichotic-reverse condition may be circumvented if an on/off switch is available on the CROS transmitter [54]. The on/off switch on a CROS transmitter could enhance speech recognition in noise, sound localization abilities, and SSQ-scores.

In summary, the analysis of the cost/effectiveness ratios for the different conditions allows us to state that the StereoBiCROS program is more interesting since it makes it possible to obtain the most significant result in the dichotic condition despite a small degradation in the reverse-dichotic condition. The Stereophonic program seems to be the one that gives the worst ratios between the two conditions but nevertheless has the advantage of never negatively degrading the SRT-50%. In any case, the fact that the patient can have access, with the same device, to the different possible stimulation modes (without limitation) is a significant advantage for them because they will be able, depending on the exposure environment, to modulate the most appropriate stimulation for its effectiveness or comfort. To date, it is the only prosthetic solution that makes it possible to have the various possible modes embedded in the same device.

The results obtained in our study on the speech intelligibility in noise of StereoBiCROS stimulation are very encouraging and are in agreement with the results obtained in Lefeuvre’s recent study on TriCROS-active [59]. We are convinced that this result is still underestimated because it is limited by the technology used. Indeed, the StereoBiCROS mode was initially intended to allow the hearing-impaired patient to hear the telephone bilaterally in their HAs, so it was diverted from its primary function. This explains why the microphone mode is necessarily fixed omnidirectional, and a certain number of speech recognition or noise filtering algorithms are not active. When it is possible to have access to additional adjustment parameters, the results could be even more significant; for example, an automatic deactivation of the CROS transfer when noise is detected on the poorer ear side (reverse-dichotic condition) would avoid degradation of the SNR in the good ear and could optimize the benefits for the hearing impaired [54]. This is why, if this mode of stimulation has already demonstrated an interest in the annoyance and intensity of tinnitus [58], other studies are necessary to assess the benefits for spatial localization [10,14,15,59] or even balance or posture disorders.

Currently, CI is considered a standard effective treatment for USNHL or ASNHL deafness [21,30,31,32,33,34,35]. Nevertheless, it is necessary to consider both the cost/effectiveness ratio and the cost/risk ratio. The estimated cost of a CI (approximately EUR 25,000) [96] is much more expensive than that of a bilateral HA (usually between EUR 2000 and 3500). The irreversibility of the CI following the possible loss of any residual hearing, surgical risks, and patients’ commitment to being involved in a rehabilitation program are all elements that must be taken into consideration. This new stimulation appears to be far less destructive than CI, and in this context, we recommend the StereoBiCROS stimulation trial for a minimum period of 30 days with ASNHL patients before considering cochlear implantation.

### 4.4. Datalogging Utilization

The study of the HA datalogging utilization is very interesting because it informs us about the subjective appreciation of the patient. With a mean utilization of over 12.4 ± 1.6 h per day, this proves that the patient experiences significant benefit from their HAs. The datalogging also reflects the subjective benefit felt between the three programs available in their daily environment. The very large majority use of the StereoBiCROS program, followed by the Stereophonic, then the BiCROS (respectively, 76.9 ± 24.2%, 18.6 ± 20.5%, and 4.5 ± 6.7%), confirms the objective results observed: the StereoBiCROS program enables performance optimizing by both ears while also optimizing speech intelligibility on the better ear via the CROS transfer. Even if we cannot confirm it, it is likely that the patient, free to change their listening mode through the different programs, used the stereophonic program when the SNR deteriorated in their better ear, i.e., in a reverse-dichotic condition. This observation would be in line with the conclusions of Kuk et al. and would be a line of thought for future studies [51].

### 4.5. QoL Questionnaire Evaluation

The results of our study for QoL demonstrate a high variability of responses to the SSQ-15 and also suggest that the majority of our ASNHL population has binaural hearing impairment, since the total score is lower than that obtained for a normal-hearing population, representing the standard [64]. Indeed, for SSQ-15, the cut-off limits (2 SD below the mean) for the normal-hearing group were 5.45 for the Speech Recognition subscale, 4.21 for the Spatial Hearing subscale, 7.63 for the Qualities of Hearing subscale, and 6.14 for the Total score. With respective scores of 3.8 ± 1.4 for Total score, 2.8 ± 1.7 for Speech score, 2.8 ± 2.0 for Spatial score, and 5.7 ± 1.9 for Qualities score, significant deficits were seen at D0 for all patients in all three subscales and total score than for the cut-off limit for normal hearing listeners (NHLs). Based on the SSQ-15 total and subscale scores, our population experienced a hearing disability. A review of the literature [18,97,98,99] suggests the same results. Similar to Dillon’s study on the effect of cochlear implantation on QoL in adults with unilateral moderate-to-profound hearing loss, we observe a significant increase in all scores (total and sub-scores), and this, only 1 month after the StereoBiCROS stimulation activation, approaching the cut-off limits for each of the scores (without exceeding them). In our study, the only subscale that exceeds the limit value at D30 is the score obtained for the dimension evaluating spatial hearing (4,8 ± 2,4 in our study versus a cut-off of 4,21 for [64]). Other authors who have used the SSQ questionnaire (in all its forms) have observed the same results with, however, some differences in the score variations or the exceeding of limit values, which could be explained by differences in age, the importance of deafness on the poor ear, or asymmetry, of the studied cohorts [98,99,100].

The results obtained in our study are therefore consistent with the literature, showing that, compared to the unaided condition, CROS systems seem to have beneficial effects on hearing-related QoL by reducing the level of listening difficulty in everyday situations [14,101]. Nevertheless, results obtained for the Short Form Health Survey (SF-12) questionnaire do not seem to be able to demonstrate that this benefit can produce effects on the improvement of the QoL related to overall health. This observation is shared in Kitterick’s meta-analysis, covering 27 studies that studied the comparative effect of different CROS on QoL for adult USNHL/ASNHL patients. The authors confirm that there is currently a lack of evidence to assess the benefit of these systems on QoL related to overall health.

### 4.6. Limitations of the Study

Some caution is required when interpreting the results of the present study. First, the relatively small sample size (N = 18) and the short duration of the trial (one month) limit the generalizability of the findings and may not fully capture long-term adaptation phenomena, which are known to occur over several months in HA fittings [102]. Furthermore, the first program provided to all participants was systematically the StereoBiCROS setting. The absence of randomization in the order of programs constitutes a potential source of bias, as initial exposure can influence subsequent preference formation and usage patterns, especially in older adults who may tend to favor the first configuration encountered.

In addition, although participants were instructed to freely switch between the three available programs and to use them in various everyday listening situations, we cannot ascertain with certainty that all individuals effectively explored each program under sufficiently diverse acoustic contexts before adopting StereoBiCROS as their predominant choice. Variability in motivation, listening habits, and real-world exposure may therefore have influenced program preference.

Another limitation concerns the modality of program switching. Participants could change programs only via the physical buttons of HAs. Given the mean age of the cohort (approximately 70 years), factors such as reduced manual dexterity, visual acuity, or cognitive processing speed—well-documented as affecting hearing-aid handling abilities [103]—may have constrained the ease and frequency of program changes. Such constraints could bias usage toward a default or more easily accessible setting rather than toward the program that would objectively provide the best benefit in a given situation.

Finally, while improvements were observed in SSQ and QoL measures after one month of HA stimulation, these global outcomes do not allow attribution of the observed benefit to any specific program among the three tested. However, since almost all participants used the HA on average 12 h per day and mostly used the StereoBiCROS program, we are convinced that the results are indeed due to the concomitant stimulation of the poorer ear with the BiCROS system during the trial.

Taken together, these limitations indicate that this study should be considered as a technical pre-validation of a promising system rather than a definitive demonstration of clinical superiority. To confirm the results obtained, it would be relevant to use Bayesian statistics. Future research should therefore include a larger number of participants, a randomized or crossover design for program order, a longer adaptation period, systematic monitoring of program use, and detailed characterization of listening environments to disentangle program-specific effects.

## 5. Conclusions

To our knowledge, the present study is the first to evaluate the performance of speech-in-noise perception in different dichotic configurations and also the evaluation of QoL with the new StereoBiCROS stimulation in a ASNHL population. Less dependent on the test condition used, and giving interesting intelligibility results in noise, this new hybrid solution appears as a credible alternative compared to other existing prosthetic solutions. It does not oblige the patient or the practitioner to choose a mode of bilateral stimulation or CROS/BiCROS. Nevertheless, if a number of methodological constraints must temper the interpretation of these results (small sample size and short trial duration, lack of randomization in the program order, etc.), we believe that this promising solution warrants further investigation to confirm its effectiveness, and that a minimum 30-day trial should be conducted before considering CI in ASNHL patients.

## Figures and Tables

**Table 1:** Patients’ characteristics. PTA: Pure Tone Average. Average thresholds at frequencies 500, 1000, 2000, 4000 Hz in dB HL (Hearing Loss). R = Right, L = Left, NR = No Response. Note: The deafness duration is the time difference (in months) between the diagnostic and the speech recognition evaluation.

# Patient	Gender	Age (Years)	Etiology	ASNHL Side	Deafness Duration (Months)	Better Ear PTA (dB HL)	Poorer Ear PTA (dB HL)
**1**	F	73	Sudden deafness (idiopathic)	R	189	31	70
**2**	F	76	Sudden deafness	R	99	68	83
**3**	M	69	Sound trauma	L	362	60	102
**4**	F	62	Sudden deafness	L	48	33	90
**5**	M	68	Sudden deafness	R	118	34	102
**6**	F	66	Sudden deafness (idiopathic)	L	66	43	89
**7**	M	71	Sudden deafness	L	109	30	85
**8**	M	70	Work in noise	L	NR	24	70
**9**	F	73	Sudden deafness	L	43	62	97
**10**	M	80	ENT history (infections)	R	72	54	70
**11**	F	80	Sudden deafness (emotional)	L	56	44	88
**12**	M	78	Work in noise	R	NR	44	94
**13**	F	83	Sudden deafness (ischemic)	L	286	48	102
**14**	F	55	ENT history (infections)	L	603	29	85
**15**	M	79	Sudden deafness (idiopathic)	R	720	49	94
**16**	M	71	Work in noise	R	NR	42	69
**17**	F	65	Sudden deafness (idiopathic)	R	40	34	77
**18**	F	54	Sudden deafness (emotional)	L	91	52	79
***Mean***		***70.7***			***193.5***	***43.4***	***85.9***
*(SD)*		*(8.2)*			*(212.4)*	*(12.5)*	*(11.5)*

**Table 2:** Description, advantages, and disadvantages of the different solutions for the rehabilitation of severe to profound AHL.

	CROS	BiCROS	Monaural HA: Poor Ear Side	Monaural HA: Better Ear Side	StereoCROS StereoBiCROS (=TriCROS)	Cochlear Implant
**Description**	-HA equipped with a microphone picking up sound from the deaf ear and transmits the signal to the contralateral ear, either in a wired or a wireless way. -Suitable for a non-fitting ear (SSD) and strictly for contralateral normal hearing.	-This is a CROS system to which a hearing aid is adapted to the better ear. -Suitable for a non-fitting ear (SSD) and/or mild to severe hearing loss for the other ear.	-Conventional HA (acoustic) fitting possible for residual hearing treatment.	-Conventional HA (acoustic) fitting if hearing loss to the better ear.	-Bilateral conventional HA (acoustic) fitting, possible even in case of interaural gap.	-Implantable medical device for restoring hearing to the SSD ear by electrical stimulation of the auditory nerve.
**Advantages**	-Fix the head-shadow effect. -Better at speech understanding in noise through dichotic or diotic conditions.	-In addition to the CROS system benefits, it compensates the hearing loss of the better ear.	-Restores binaurality. -Improves sound localizations. -Contributes to the treatment of tinnitus on the poor side (if associated).	-Compensates only the hearing loss.	-Restores binaurality. -Improves sound localizations. -Contributes to the treatment of tinnitus of the poor side (if associated).	-Unlike BAHA and CROS that bypass deafness by transmitting the signal to the contralateral ear, cochlear implant treats hearing loss and allows restoration of binaural mechanisms (head-shadow effect, summation effect, binaural, and Squelch effect. -Restores real binaurality. -Improves sound localization and speech discrimination in noise.
**Disadvantages**	-Does not restore binaural hearing. -Only sound capture of the deaf without any hearing restoration. -Degradation of speech discrimination in noise in reverse-dichotic condition. -Does not allow sound localization. -Does not treat tinnitus of the deaf ear, and is sometimes more detrimental.	-Identical to the CROS system.	-Only possible if sufficient residual hearing but also in the presence of a limited interaural gap allowing for stereoacoustic stimulation. -Limited results in residual capacity to the poorer ear. -Challenging fitting.	-Does not restore binaural hearing. -Definitely condemns the poor ear.	-Only possible if residual hearing sufficient but also in the presence of a limited interaural gap allowing for stereoacoustic stimulation. -Difficult fitting.	-No reimbursement from Health Insurance. -Requires surgery. -Insufficient level of clinical evidence.

## Data Availability

The datasets generated and analyzed during the current study are not publicly available due to privacy and ethical restrictions but are available from the first author, Dr. Morgan Potier, upon reasonable request.

## Abbreviations

**Acronym**	**Signification**
AC	Air Conduction
ANSI	American National Standards Institute
ASNHL	Asymmetric Sensorineural Hearing Loss
BAHA	Bone-Anchored Hearing Aid
BC	Bone Conduction
BiCROS	Bilateral Contralateral Routing of Signal
CI	Cochlear Implant
CPP	Comité de Protection des Personnes
CROS	Contralateral Routing of Signal
dB	deciBel
HA	Hearing Aid
HL	Hearing Level
ILD	Interaural Level Difference
ISO	International Organization for Standardization
ITD	Interaural Time Difference
PTA	Pure Tone Average
QoL	Quality of Life
REM	Real Ear Measurement
SF-12	12-Item Short Form Health Survey
SF-36	36-Item Short Form Health Survey
SNR	Signal-to-Noise Ratio
SPL	Sound Pressure Level
SRT	Speech Recognition Thresholds
SSD	Single-Sided Deafness
SSQ	Speech, Spatial and Qualities of Hearing Scale
StereoBiCROS	BiCROS with bilateral amplification (stereophonic)
USNHL	Unilateral Sensorineural Hearing Loss
